# Supplementary material for: Diverse Co(II) Coordination Polymers with the Same Mixed Ligands: Evaluation of Chemical Stability and Structural Transformation
Source: Molecules. 2024 Apr 12;29(8):1748. doi: 10.3390/molecules29081748 (PMC11051802; doi:10.3390/molecules29081748)

checkCIF/PLATON report

Structure factors have been supplied for datablock(s) 1, 2, 3, 4

THIS REPORT IS FOR GUIDANCE ONLY. IF USED AS PART OF A REVIEW PROCEDURE FOR PUBLICATION, IT SHOULD NOT REPLACE THE EXPERTISE OF AN EXPERIENCED CRYSTALLOGRAPHIC REFEREE.

No syntax errors found. CIF dictionary Interpreting this report

Datablock: 1

Bond precision: C-C = 0.0030 A Wavelength=0.71073

Cell:	a=12.0281(8)	b=12.3628(9)	c=12.5821(9)
	alpha=110.490(3)	beta=99.692(3)	gamma=108.754(3)
Temperature:	296 K		

	Calculated	Reported
Volume	1574.1(2)	1574.1(2)
Space group	P -1	P -1
Hall group	-P 1	-P 1
Moiety formula	C28 H26 Co N4 O10 S, C H4 O, H2 O	?
Sum formula	C29 H32 Co N4 O12 S	C29 H32 Co N4 O12 S
Mr	719.58	719.57
Dx,g cm-3	1.518	1.518
Z	2	2
Mu (mm-1)	0.682	0.682
F000	746.0	746.0
F000'	747.32	
h,k,lmax	16,16,16	16,16,16
Nref	7897	7756
Tmin,Tmax	0.782,0.872	0.660,0.746
Tmin'	0.711	

Correction method= # Reported T Limits: Tmin=0.660 Tmax=0.746
AbsCorr = MULTI_SCAN

Data completeness= 0.982 Theta(max)= 28.390

R(reflections)= 0.0413(7237) wR2(reflections)= 0.1089(7756)

S = 1.048 Npar= 452

The following ALERTS were generated. Each ALERT has the format
test-name_ALERT_alert-type_alert-level.
Click on the hyperlinks for more details of the test.

Alert level C

```
PLAT250_ALERT_2_C Large U3/U1 Ratio for <U(i,j)> Tensor(Resd    1)        2.1 Note
PLAT369_ALERT_2_C Long   C(sp2)-C(sp2) Bond  C7       - C8       .       1.54 Ang.
PLAT911_ALERT_3_C Missing FCF Refl Between Thmin & STh/L=    0.600          6 Report
                6 -9 10,   4-11 12,   5-11 12,   3-11 13,   2-10 13,   1 -9 14,
PLAT971_ALERT_2_C Check Calcd Resid. Dens.  1.17Ang From O10             1.62 eA-3
PLAT975_ALERT_2_C Check Calcd Resid. Dens.  0.71Ang From O10     .       0.73 eA-3
PLAT976_ALERT_2_C Check Calcd Resid. Dens.  0.88Ang From O10     .      -1.04 eA-3
PLAT976_ALERT_2_C Check Calcd Resid. Dens.  0.96Ang From O10     .      -0.57 eA-3
PLAT977_ALERT_2_C Check Negative Difference Density on H10A      .      -0.48 eA-3
PLAT977_ALERT_2_C Check Negative Difference Density on H10B      .      -0.79 eA-3
PLAT977_ALERT_2_C Check Negative Difference Density on H12E      .      -0.72 eA-3
PLAT977_ALERT_2_C Check Negative Difference Density on H29A      .      -0.34 eA-3
PLAT977_ALERT_2_C Check Negative Difference Density on H29B      .      -0.44 eA-3
PLAT977_ALERT_2_C Check Negative Difference Density on H29D      .      -0.36 eA-3
PLAT977_ALERT_2_C Check Negative Difference Density on H29E      .      -0.31 eA-3
PLAT977_ALERT_2_C Check Negative Difference Density on H29F      .      -0.52 eA-3
```

Alert level G

```
PLAT004_ALERT_5_G Polymeric Structure Found with Maximum Dimension          2 Info
PLAT007_ALERT_5_G Number of Unrefined Donor-H Atoms .............          11 Report
              H2A    H3A    H9C    H9D    H10A   H10B   H12E   H11B   H11C   H11E   H11F
PLAT154_ALERT_1_G The s.u.'s on the Cell Angles are Equal ..(Note)      0.003 Degree
PLAT300_ALERT_4_G Atom Site Occupancy of O12        Constrained at        0.5 Check
PLAT300_ALERT_4_G Atom Site Occupancy of O12'       Constrained at        0.5 Check
PLAT300_ALERT_4_G Atom Site Occupancy of C29        Constrained at        0.5 Check
PLAT300_ALERT_4_G Atom Site Occupancy of C29'       Constrained at        0.5 Check
PLAT300_ALERT_4_G Atom Site Occupancy of H29A       Constrained at        0.5 Check
PLAT300_ALERT_4_G Atom Site Occupancy of H29B       Constrained at        0.5 Check
PLAT300_ALERT_4_G Atom Site Occupancy of H29C       Constrained at        0.5 Check
PLAT300_ALERT_4_G Atom Site Occupancy of H29D       Constrained at        0.5 Check
PLAT300_ALERT_4_G Atom Site Occupancy of H29E       Constrained at        0.5 Check
PLAT300_ALERT_4_G Atom Site Occupancy of H29F       Constrained at        0.5 Check
PLAT302_ALERT_4_G Anion/Solvent/Minor-Residue Disorder (Resd    2)       100% Note
PLAT302_ALERT_4_G Anion/Solvent/Minor-Residue Disorder (Resd    3)       100% Note
PLAT302_ALERT_4_G Anion/Solvent/Minor-Residue Disorder (Resd    4)       100% Note
PLAT309_ALERT_2_G Single Bonded Oxygen (C-O > 1.3 Ang) ..........        O12' Check
PLAT413_ALERT_2_G Short Inter XH3 .. XHn     H6C      ..H29F     .       2.06 Ang.
                                               2-x,2-y,2-z  =      2_777 Check
PLAT413_ALERT_2_G Short Inter XH3 .. XHn     H18A     ..H29E     .       1.75 Ang.
                                               3-x,2-y,2-z  =      2_877 Check
PLAT414_ALERT_2_G Short Intra D-H..H-X       H12E     ..H29A     .       2.11 Ang.
                                                   x,y,z  =      1_555 Check
PLAT414_ALERT_2_G Short Intra D-H..H-X       H12E     ..H29D     .       1.69 Ang.
                                                   x,y,z  =      1_555 Check
PLAT414_ALERT_2_G Short Intra D-H..H-X       H12E     ..H29E     .       1.91 Ang.
                                                   x,y,z  =      1_555 Check
PLAT414_ALERT_2_G Short Intra D-H..H-X       H12E     ..H29F     .       0.40 Ang.
                                                   x,y,z  =      1_555 Check
PLAT417_ALERT_2_G Short Inter D-H..H-D       H3A      ..H11F     .       1.35 Ang.
```

```
                                                    x,y,1+z  =      1_556 Check
PLAT417_ALERT_2_G Short Inter D-H..H-D       H10B     ..H11B     .       1.97 Ang.
                                                2-x,2-y,1-z  =      2_776 Check
PLAT804_ALERT_5_G Number of ARU-Code Packing Problem(s) in PLATON          8 Info
PLAT883_ALERT_1_G No Info/Value for _atom_sites_solution_primary .     Please Do !
PLAT912_ALERT_4_G Missing # of FCF Reflections Above STh/L=  0.600       135 Note
PLAT941_ALERT_3_G Average HKL Measurement Multiplicity ..........        3.6 Low
PLAT969_ALERT_5_G The 'Henn et al.' R-Factor-gap value ..........       4.05 Note
              Predicted wR2: Based on SigI**2  2.69 or SHELX Weight 10.70
PLAT978_ALERT_2_G Number C-C Bonds with Positive Residual Density.         9 Info
PLAT992_ALERT_5_G Repd & Actual _reflns_number_gt Values Differ by         2 Check
```

```
   0 ALERT level A = Most likely a serious problem - resolve or explain
   0 ALERT level B = A potentially serious problem, consider carefully
  15 ALERT level C = Check. Ensure it is not caused by an omission or oversight
  32 ALERT level G = General information/check it is not something unexpected

   2 ALERT type 1 CIF construction/syntax error, inconsistent or missing data
  24 ALERT type 2 Indicator that the structure model may be wrong or deficient
   2 ALERT type 3 Indicator that the structure quality may be low
  14 ALERT type 4 Improvement, methodology, query or suggestion
   5 ALERT type 5 Informative message, check
```

Datablock: 2

Bond precision:	C-C = 0.0024 A		Wavelength=0.71073
Cell:	a=10.537(3)	b=12.378(2)	c=12.849(2)
	alpha=61.476(8)	beta=72.697(10)	gamma=85.374(14)
Temperature:	293 K		

	Calculated	Reported
Volume	1402.0(5)	1402.0(5)
Space group	P -1	P -1
Hall group	-P 1	-P 1
Moiety formula	C21 H15 Co N2 O7 S, C7 H7 N2 O, 2(H2 O)	?
Sum formula	C28 H26 Co N4 O10 S	C28 H26 Co N4 O10 S
Mr	669.52	669.52
Dx,g cm-3	1.586	1.586
Z	2	2
Mu (mm-1)	0.754	0.754
F000	690.0	690.0
F000'	691.27	
h,k,lmax	14,16,17	14,16,17
Nref	6953	6938
Tmin,Tmax	0.834,0.860	0.685,0.746
Tmin'	0.740	

Correction method= # Reported T Limits: Tmin=0.685 Tmax=0.746
AbsCorr = MULTI_SCAN

Data completeness= 0.998 Theta(max)= 28.280

R(reflections)= 0.0296(6419) wR2(reflections)= 0.0841(6938)

S = 1.063 Npar= 397

The following ALERTS were generated. Each ALERT has the format
test-name_ALERT_alert-type_alert-level.
Click on the hyperlinks for more details of the test.

Alert level C

```
PLAT245_ALERT_2_C U(iso) H9C       Smaller than U(eq) O9        by      0.042 Ang**2
PLAT245_ALERT_2_C U(iso) H9D       Smaller than U(eq) O9        by      0.042 Ang**2
PLAT369_ALERT_2_C Long   C(sp2)-C(sp2) Bond  C7       - C7_e     .       1.54 Ang.
PLAT369_ALERT_2_C Long   C(sp2)-C(sp2) Bond  C28      - C28_f    .       1.54 Ang.
PLAT911_ALERT_3_C Missing FCF Refl Between Thmin & STh/L=    0.600          2 Report
               5 -3 10,   5 10 14,
PLAT976_ALERT_2_C Check Calcd Resid. Dens.  0.65Ang From O9      .      -0.94 eA-3
PLAT976_ALERT_2_C Check Calcd Resid. Dens.  0.58Ang From O9      .      -0.88 eA-3
PLAT976_ALERT_2_C Check Calcd Resid. Dens.  0.48Ang From O9      .      -0.87 eA-3
PLAT976_ALERT_2_C Check Calcd Resid. Dens.  0.46Ang From O9      .      -0.85 eA-3
PLAT977_ALERT_2_C Check Negative Difference Density on H9C       .      -0.42 eA-3
PLAT977_ALERT_2_C Check Negative Difference Density on H9D       .      -0.52 eA-3
```

Alert level G

```
PLAT004_ALERT_5_G Polymeric Structure Found with Maximum Dimension          2 Info
PLAT007_ALERT_5_G Number of Unrefined Donor-H Atoms .............           6 Report
              H2A   H4A   H9C   H9D   H10C  H10D
PLAT199_ALERT_1_G Reported _cell_measurement_temperature ..... (K)        293 Check
PLAT200_ALERT_1_G Reported   _diffrn_ambient_temperature ..... (K)        293 Check
PLAT794_ALERT_5_G Tentative Bond Valency for Co        (II)      .       1.98 Info
PLAT804_ALERT_5_G Number of ARU-Code Packing Problem(s) in PLATON           1 Info
PLAT883_ALERT_1_G No Info/Value for _atom_sites_solution_primary .     Please Do !
PLAT912_ALERT_4_G Missing # of FCF Reflections Above STh/L=  0.600         13 Note
PLAT941_ALERT_3_G Average HKL Measurement Multiplicity ..........         3.7 Low
PLAT969_ALERT_5_G The 'Henn et al.' R-Factor-gap value ..........        4.45 Note
              Predicted wR2: Based on SigI**2  1.89 or SHELX Weight  8.14
PLAT978_ALERT_2_G Number C-C Bonds with Positive Residual Density.         20 Info
```

---

0 **ALERT level A** = Most likely a serious problem - resolve or explain
0 **ALERT level B** = A potentially serious problem, consider carefully
11 **ALERT level C** = Check. Ensure it is not caused by an omission or oversight
11 **ALERT level G** = General information/check it is not something unexpected

3 ALERT type 1 CIF construction/syntax error, inconsistent or missing data
11 ALERT type 2 Indicator that the structure model may be wrong or deficient
2 ALERT type 3 Indicator that the structure quality may be low
1 ALERT type 4 Improvement, methodology, query or suggestion
5 ALERT type 5 Informative message, check

Datablock: 3

Bond precision: C-C = 0.0055 A Wavelength=0.71073

Cell:	a=11.6913(8)	b=12.5564(8)	c=13.2862(8)
	alpha=105.7159(18)	beta=105.6037(18)	gamma=91.3885(19)
Temperature:	296 K		

	Calculated	Reported
Volume	1798.4(2)	1798.4(2)
Space group	P -1	P -1
Hall group	-P 1	-P 1
Moiety formula	C35 H31 Co N6 O10 S, O	?
Sum formula	C35 H31 Co N6 O11 S	C35 H33 Co N6 O11 S
Mr	802.65	804.66
Dx,g cm-3	1.482	1.486
Z	2	2
Mu (mm-1)	0.605	0.605
F000	828.0	832.0
F000’	829.33	
h,k,lmax	14,15,16	14,15,16
Nref	7084	7082
Tmin,Tmax	0.930,0.941	
Tmin’	0.886	

Correction method= Not given

Data completeness= 1.000 Theta(max)= 26.000

R(reflections)= 0.0535(4176) wR2(reflections)= 0.1061(7082)

S = 1.004 Npar= 497

The following ALERTS were generated. Each ALERT has the format
test-name_ALERT_alert-type_alert-level.
Click on the hyperlinks for more details of the test.

Alert level C

```
PLAT041_ALERT_1_C Calc. and Reported SumFormula    Strings  Differ     Please Check
             Calc: C35 H31 Co N6 O11 S
             Rep.: C35 H33 Co N6 O11 S
PLAT043_ALERT_1_C Calculated and Reported Mol. Weight Differ by ..       2.01 Check
PLAT068_ALERT_1_C Reported F000 Differs from Calcd (or Missing)...     Please Check
PLAT242_ALERT_2_C Low    'MainMol' Ueq as Compared to Neighbors of        C29 Check
PLAT260_ALERT_2_C Large Average Ueq of Residue Including      O11'      0.103 Check
PLAT369_ALERT_2_C Long   C(sp2)-C(sp2) Bond  C7       - C8       .       1.53 Ang.
PLAT369_ALERT_2_C Long   C(sp2)-C(sp2) Bond  C21      - C21_e    .       1.53 Ang.
PLAT906_ALERT_3_C Large K Value in the Analysis of Variance ......      2.942 Check
```

Alert level G

```
FORMU01_ALERT_2_G  There is a discrepancy between the atom counts in the
            _chemical_formula_sum and the formula from the _atom_site* data.
            Atom count from _chemical_formula_sum:C35 H33 Co1 N6 O11 S1
            Atom count from the _atom_site data:  C35 H31 Co1 N6 O11 S1
CELLZ01_ALERT_1_G Difference between formula and atom_site contents detected.
```

```
CELLZ01_ALERT_1_G WARNING: H atoms missing from atom site list. Is this intentional?
           From the CIF: _cell_formula_units_Z    2
           From the CIF: _chemical_formula_sum  C35 H33 Co N6 O11 S
           TEST: Compare cell contents of formula and atom_site data

           atom    Z*formula  cif sites diff
           C         70.00     70.00    0.00
           H         66.00     62.00    4.00
           Co         2.00      2.00    0.00
           N         12.00     12.00    0.00
           O         22.00     22.00    0.00
           S          2.00      2.00    0.00
PLAT004_ALERT_5_G Polymeric Structure Found with Maximum Dimension          2 Info
PLAT007_ALERT_5_G Number of Unrefined Donor-H Atoms .............          5 Report
              H2A   H3A   H6A   H10A  H10C
PLAT302_ALERT_4_G Anion/Solvent/Minor-Residue Disorder (Resd    2)       100% Note
PLAT302_ALERT_4_G Anion/Solvent/Minor-Residue Disorder (Resd    3)       100% Note
PLAT311_ALERT_2_G Isolated Disordered Oxygen Atom (No H's ?) .....        O11 Check
PLAT311_ALERT_2_G Isolated Disordered Oxygen Atom (No H's ?) .....       O11' Check
PLAT794_ALERT_5_G Tentative Bond Valency for Co        (II)      .       1.91 Info
PLAT883_ALERT_1_G No Info/Value for _atom_sites_solution_primary .     Please Do !
PLAT899_ALERT_4_G SHELXL2018 is Deprecated and Succeeded by SHELXL     2019/3 Note
PLAT967_ALERT_5_G Note: Two-Theta Cutoff Value in Embedded .res ..       52.0 Degree
PLAT969_ALERT_5_G The 'Henn et al.' R-Factor-gap value ..........       1.89 Note
              Predicted wR2: Based on SigI**2  5.62 or SHELX Weight 10.97
PLAT978_ALERT_2_G Number C-C Bonds with Positive Residual Density.          0 Info
```

---

   0 **ALERT level A** = Most likely a serious problem - resolve or explain
   0 **ALERT level B** = A potentially serious problem, consider carefully
   8 **ALERT level C** = Check. Ensure it is not caused by an omission or oversight
  15 **ALERT level G** = General information/check it is not something unexpected

   6 ALERT type 1 CIF construction/syntax error, inconsistent or missing data
   8 ALERT type 2 Indicator that the structure model may be wrong or deficient
   1 ALERT type 3 Indicator that the structure quality may be low
   3 ALERT type 4 Improvement, methodology, query or suggestion
   5 ALERT type 5 Informative message, check

Datablock: 4

Bond precision: C-C = 0.0070 A   Wavelength=0.71073

Cell:	a=13.9967(9)	b=14.4949(8)	c=14.9094(8)
	alpha=104.0893(14)	beta=93.1620(15)	gamma=114.1365(14)
Temperature:	296 K		

	Calculated	Reported
Volume	2636.1(3)	2636.1(3)
Space group	P -1	P -1
Hall group	-P 1	-P 1
Moiety formula	C49 H40 Co2 N6 O17 S2, 3(H2 O), O	?
Sum formula	C49 H46 Co2 N6 O21 S2	C49 H49 Co2 N6 O21 S2
Mr	1236.90	1239.92
Dx,g cm-3	1.558	1.562
Z	2	2
Mu (mm-1)	0.795	0.795
F000	1272.0	1278.0
F000’	1274.53	
h,k,lmax	17,17,18	17,17,18
Nref	10361	10361
Tmin,Tmax	0.735,0.728	0.681,0.746
Tmin’	0.720	

Correction method= # Reported T Limits: Tmin=0.681 Tmax=0.746
AbsCorr = MULTI_SCAN

Data completeness= 1.000 Theta(max)= 25.999

R(reflections)= 0.0495(7417) wR2(reflections)= 0.1364(10361)

S = 1.019 Npar= 776

The following ALERTS were generated. Each ALERT has the format
test-name_ALERT_alert-type_alert-level.
Click on the hyperlinks for more details of the test.

Alert level C

```
PLAT041_ALERT_1_C Calc. and Reported SumFormula    Strings  Differ     Please Check
              Calc: C49 H46 Co2 N6 O21 S2
              Rep.: C49 H49 Co2 N6 O21 S2
PLAT042_ALERT_1_C Calc. and Reported MoietyFormula Strings  Differ     Please Check
              Calc: C49 H40 Co2 N6 O17 S2, 3(H2 O), O
              Rep.: ?’
                                                            ’
PLAT043_ALERT_1_C Calculated and Reported Mol. Weight Differ by ..       3.02 Check
PLAT068_ALERT_1_C Reported F000 Differs from Calcd (or Missing)...     Please Check
PLAT202_ALERT_3_C Isotropic non-H Atoms in Anion/Solvent ........          1 Check
              O21
PLAT213_ALERT_2_C Atom N6              has ADP max/min Ratio .....        3.3 prolat
PLAT213_ALERT_2_C Atom C48             has ADP max/min Ratio .....        3.3 prolat
PLAT213_ALERT_2_C Atom C49             has ADP max/min Ratio .....        3.4 prolat
PLAT220_ALERT_2_C NonSolvent   Resd 1  C   Ueq(max)/Ueq(min) Range        4.9 Ratio
PLAT220_ALERT_2_C NonSolvent   Resd 1  N   Ueq(max)/Ueq(min) Range        3.2 Ratio
```

PLAT241_ALERT_2_C High 'MainMol' Ueq as Compared to Neighbors of N6 Check
PLAT241_ALERT_2_C High 'MainMol' Ueq as Compared to Neighbors of C21 Check
PLAT241_ALERT_2_C High 'MainMol' Ueq as Compared to Neighbors of C48 Check
PLAT241_ALERT_2_C High 'MainMol' Ueq as Compared to Neighbors of C49 Check
PLAT242_ALERT_2_C Low 'MainMol' Ueq as Compared to Neighbors of C44 Check
PLAT242_ALERT_2_C Low 'MainMol' Ueq as Compared to Neighbors of C47 Check
PLAT260_ALERT_2_C Large Average Ueq of Residue Including O20 0.140 Check
PLAT260_ALERT_2_C Large Average Ueq of Residue Including O19 0.164 Check
PLAT329_ALERT_4_C Carbon Atom Hybridisation Unclear for .... C20' Check
PLAT334_ALERT_2_C Small <C-C> Benzene Dist. C44 -C49 . 1.37 Ang.
PLAT341_ALERT_3_C Low Bond Precision on C-C Bonds .... 0.00695 Ang.
PLAT369_ALERT_2_C Long C(sp2)-C(sp2) Bond C7 - C8 . 1.53 Ang.
PLAT417_ALERT_2_C Short Inter D-H..H-D H17C ..H19C . 2.11 Ang.
1+x,y,z = 1_655 Check
PLAT905_ALERT_3_C Negative K value in the Analysis of Variance ... -2.112 Report
PLAT976_ALERT_2_C Check Calcd Resid. Dens. 0.75Ang From O19 . -0.59 eA-3
PLAT976_ALERT_2_C Check Calcd Resid. Dens. 0.75Ang From O19 . -0.42 eA-3
PLAT976_ALERT_2_C Check Calcd Resid. Dens. 0.67Ang From O19 . -0.42 eA-3
PLAT976_ALERT_2_C Check Calcd Resid. Dens. 0.96Ang From O17 . -0.41 eA-3

Alert level G

FORMU01_ALERT_2_G There is a discrepancy between the atom counts in the
_chemical_formula_sum and the formula from the _atom_site* data.
Atom count from _chemical_formula_sum:C49 H49 Co2 N6 O21 S2
Atom count from the _atom_site data: C49 H46 Co2 N6 O21 S2
CELLZ01_ALERT_1_G Difference between formula and atom_site contents detected.
CELLZ01_ALERT_1_G WARNING: H atoms missing from atom site list. Is this intentional?
From the CIF: _cell_formula_units_Z 2
From the CIF: _chemical_formula_sum C49 H49 Co2 N6 O21 S2
TEST: Compare cell contents of formula and atom_site data

atom	Z*formula	cif sites	diff
C	98.00	98.00	0.00
H	98.00	92.00	6.00
Co	4.00	4.00	0.00
N	12.00	12.00	0.00
O	42.00	42.00	0.00
S	4.00	4.00	0.00

PLAT002_ALERT_2_G Number of Distance or Angle Restraints on AtSite 3 Note
PLAT003_ALERT_2_G Number of Uiso or Uij Restrained non-H Atoms ... 1 Report
PLAT004_ALERT_5_G Polymeric Structure Found with Maximum Dimension 2 Info
PLAT007_ALERT_5_G Number of Unrefined Donor-H Atoms .... 14 Report
H2A H3A H16B H16C H17C H17D H20E H20F H18A H18B H19C
H19D H18D H18E
PLAT083_ALERT_2_G SHELXL Second Parameter in WGHT Unusually Large 5.86 Why ?
PLAT172_ALERT_4_G The CIF-Embedded .res File Contains DFIX Records 1 Report
PLAT301_ALERT_3_G Main Residue Disorder ....(Resd 1) 5% Note
PLAT302_ALERT_4_G Anion/Solvent/Minor-Residue Disorder (Resd 2) 100% Note
PLAT302_ALERT_4_G Anion/Solvent/Minor-Residue Disorder (Resd 3) 100% Note
PLAT302_ALERT_4_G Anion/Solvent/Minor-Residue Disorder (Resd 5) 100% Note
PLAT302_ALERT_4_G Anion/Solvent/Minor-Residue Disorder (Resd 6) 100% Note
PLAT302_ALERT_4_G Anion/Solvent/Minor-Residue Disorder (Resd 7) 100% Note
PLAT311_ALERT_2_G Isolated Disordered Oxygen Atom (No H's ?) ..... O20' Check
PLAT311_ALERT_2_G Isolated Disordered Oxygen Atom (No H's ?) ..... O21 Check
PLAT311_ALERT_2_G Isolated Disordered Oxygen Atom (No H's ?) ..... O21' Check
PLAT410_ALERT_2_G Short Intra H...H Contact H48A ..H19B . 2.01 Ang.

```
                                                    x,y,-1+z  =      1_554 Check
PLAT414_ALERT_2_G Short Intra D-H..H-X       H17C     ..H19A     .       1.92 Ang.
                                                       x,y,z  =      1_555 Check
PLAT417_ALERT_2_G Short Inter D-H..H-D       H3A      ..H18A     .       1.53 Ang.
                                                       x,y,z  =      1_555 Check
PLAT794_ALERT_5_G Tentative Bond Valency for Co1       (II)      .       1.98 Info
PLAT860_ALERT_3_G Number of Least-Squares Restraints ............          2 Note
PLAT883_ALERT_1_G No Info/Value for _atom_sites_solution_primary .     Please Do !
PLAT941_ALERT_3_G Average HKL Measurement Multiplicity ...........        4.5 Low
PLAT965_ALERT_2_G The SHELXL WEIGHT Optimisation has not Converged     Please Check
PLAT967_ALERT_5_G Note: Two-Theta Cutoff Value in Embedded .res ..       52.0 Degree
PLAT969_ALERT_5_G The 'Henn et al.' R-Factor-gap value ..........       3.40 Note
              Predicted wR2: Based on SigI**2  4.01 or SHELX Weight 13.91
PLAT978_ALERT_2_G Number C-C Bonds with Positive Residual Density.          0 Info
```

---

0 **ALERT level A** = Most likely a serious problem - resolve or explain
0 **ALERT level B** = A potentially serious problem, consider carefully
28 **ALERT level C** = Check. Ensure it is not caused by an omission or oversight
29 **ALERT level G** = General information/check it is not something unexpected

7 ALERT type 1 CIF construction/syntax error, inconsistent or missing data
32 ALERT type 2 Indicator that the structure model may be wrong or deficient
6 ALERT type 3 Indicator that the structure quality may be low
7 ALERT type 4 Improvement, methodology, query or suggestion
5 ALERT type 5 Informative message, check

It is advisable to attempt to resolve as many as possible of the alerts in all categories. Often the minor alerts point to easily fixed oversights, errors and omissions in your CIF or refinement strategy, so attention to these fine details can be worthwhile. In order to resolve some of the more serious problems it may be necessary to carry out additional measurements or structure refinements. However, the purpose of your study may justify the reported deviations and the more serious of these should normally be commented upon in the discussion or experimental section of a paper or in the "special_details" fields of the CIF. checkCIF was carefully designed to identify outliers and unusual parameters, but every test has its limitations and alerts that are not important in a particular case may appear. Conversely, the absence of alerts does not guarantee there are no aspects of the results needing attention. It is up to the individual to critically assess their own results and, if necessary, seek expert advice.

Publication of your CIF in IUCr journals

A basic structural check has been run on your CIF. These basic checks will be run on all CIFs submitted for publication in IUCr journals (*Acta Crystallographica*, *Journal of Applied Crystallography*, *Journal of Synchrotron Radiation*); however, if you intend to submit to *Acta Crystallographica Section C* or *E* or *IUCrData*, you should make sure that full publication checks are run on the final version of your CIF prior to submission.

Publication of your CIF in other journals

Please refer to the *Notes for Authors* of the relevant journal for any special instructions relating to CIF submission.

PLATON version of 06/01/2024; check.def file version of 05/01/2024

Datablock 1 - ellipsoid plot

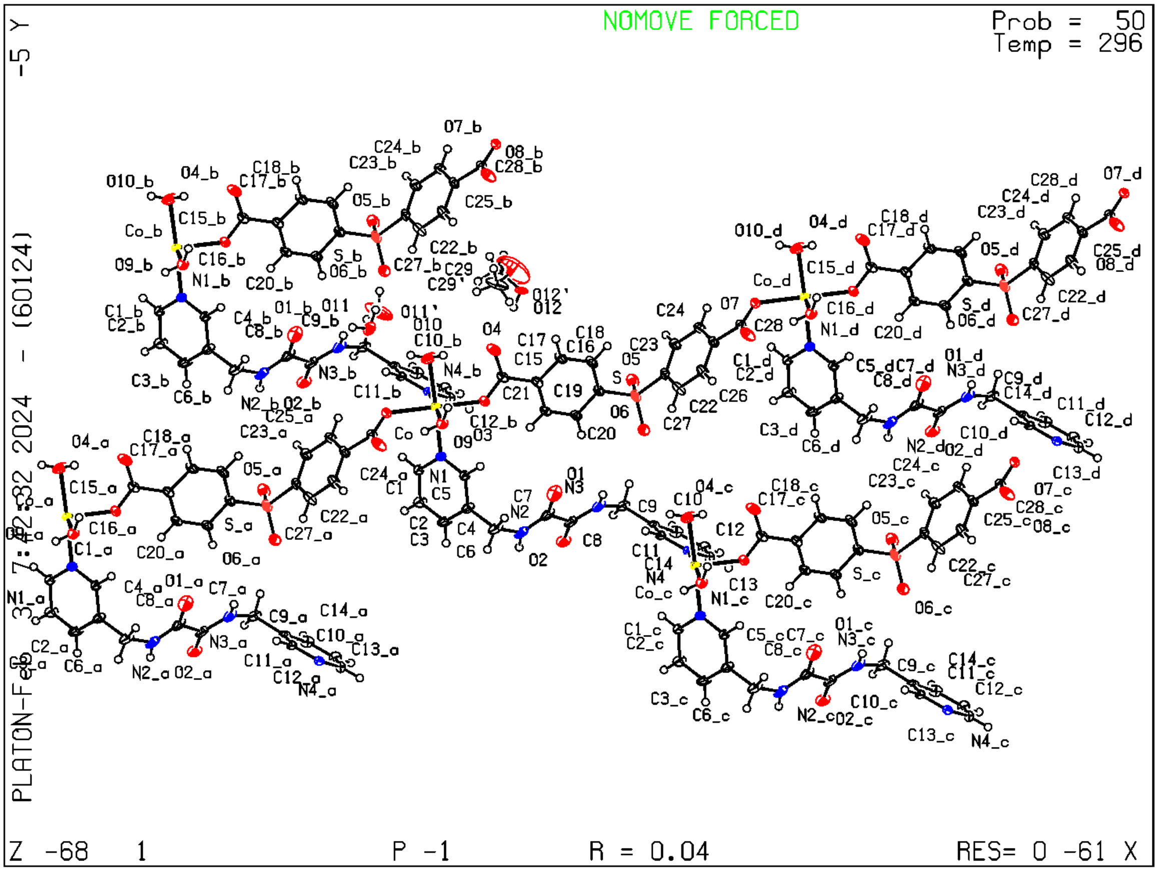

Datablock 2 - ellipsoid plot

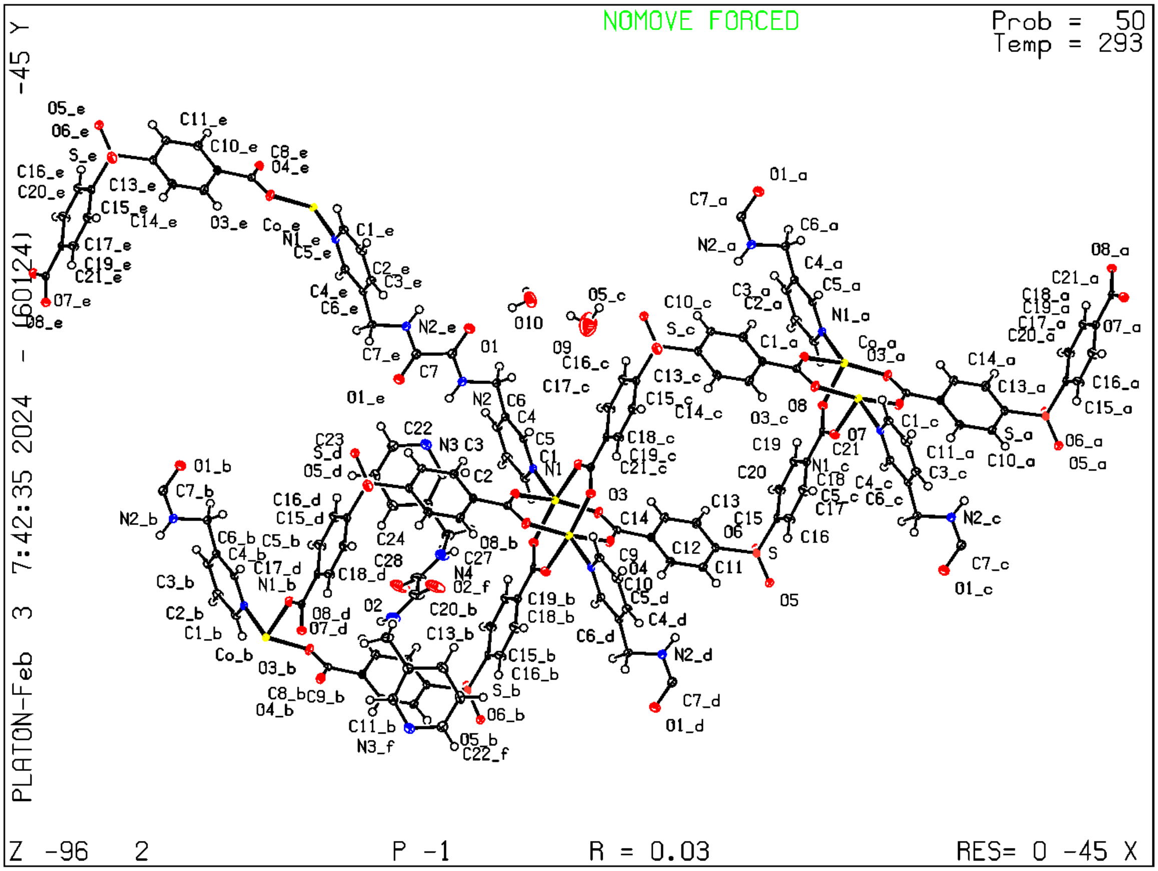

Datablock 3 - ellipsoid plot

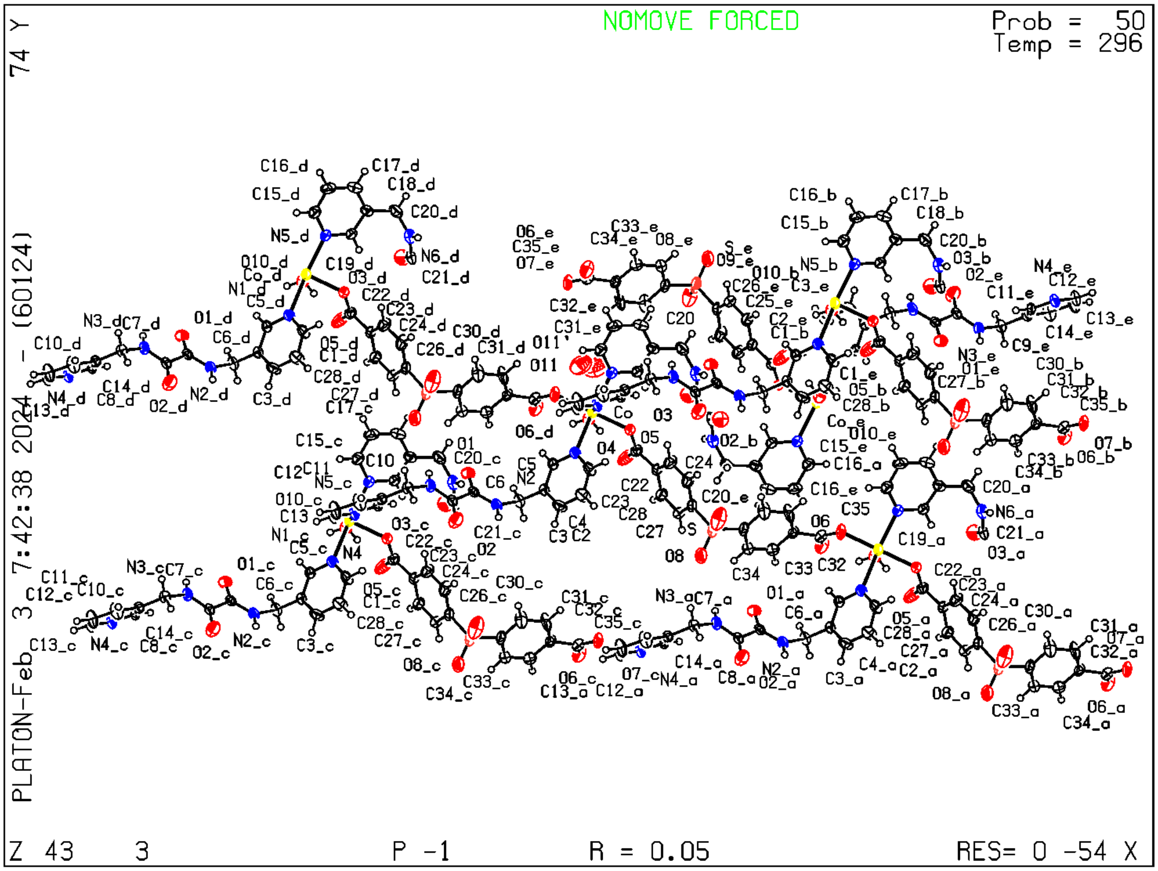

Datablock 4 - ellipsoid plot

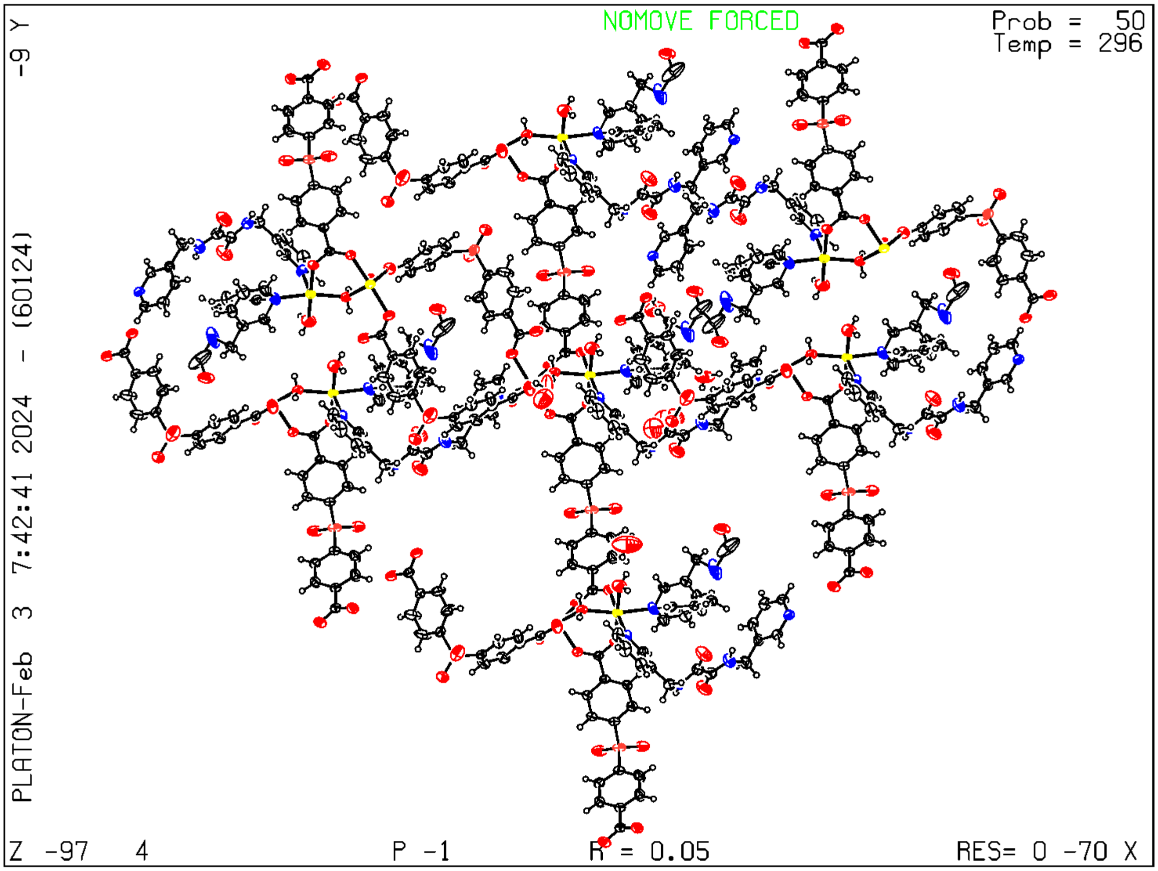

Supplement: Supplementary file 1 [file molecules-29-01748-s001.zip › checkcif.pdf]
