# Supplementary material for: Diverse Co(II) Coordination Polymers with the Same Mixed Ligands: Evaluation of Chemical Stability and Structural Transformation
Source: Molecules. 2024 Apr 12;29(8):1748. doi: 10.3390/molecules29081748 (PMC11051802; doi:10.3390/molecules29081748)

Supplementary Materials

Diverse Co(II) Coordination Polymers with Same Mixed Ligands: Evaluation for Chemical Stability and Structural Transformation

Chia-Yi Lee,[1] Yu-Hui Ye,[1] Song-Wei Wang[1] and Jhy-Der Chen[1,*]

[1] Department of Chemistry, Chung Yuan Christian University, Chung Li, Taoyuan City 320, Taiwan; miss10031031@gmail.com (C.-Y.L.); a4862225@gmail.com (Y.-H. Y.); bvbv20520@gmail.com (S.-W.W.)

* Correspondence: jdchen@cycu.edu.tw (J.-D.C.); Tel.: +886-3-265-3351 (J.-D.C.)

Figure S1. The PXRD patterns of complex **1**.

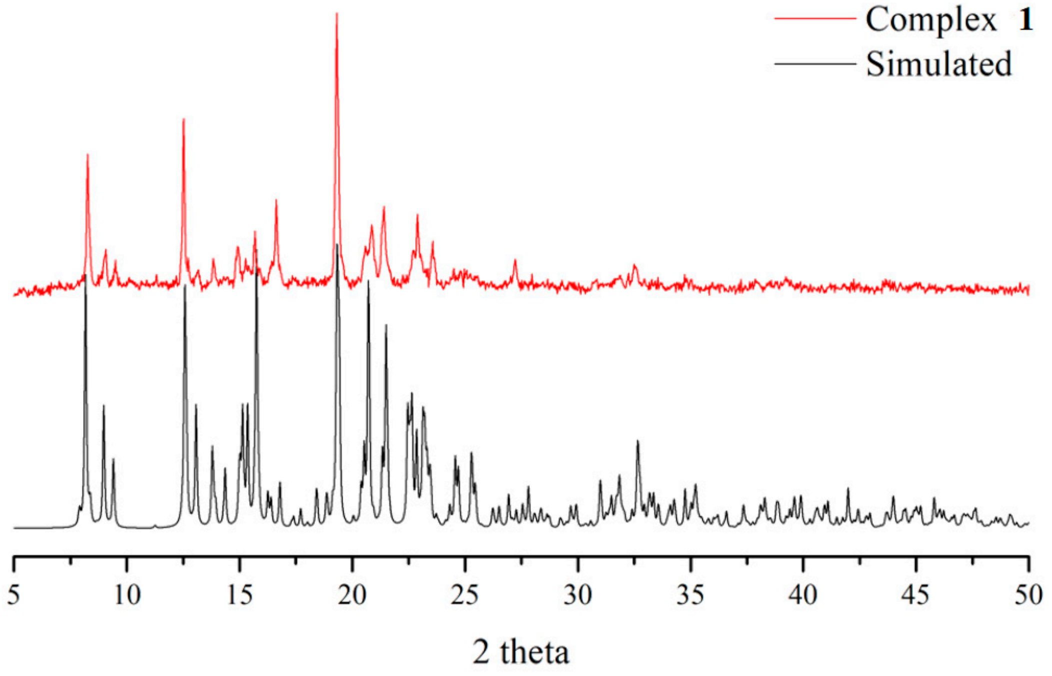

Figure S2. The PXRD patterns of complex **2**.

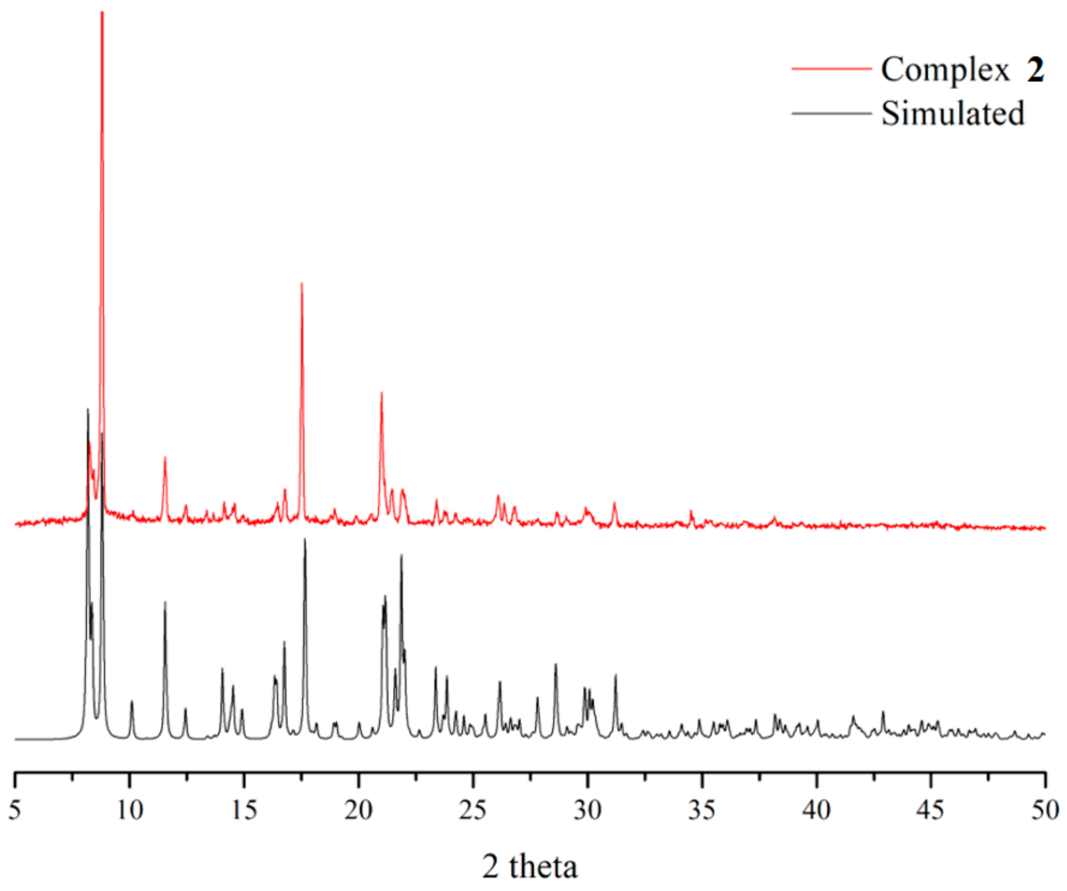

Figure S3. The PXRD patterns of complex **3**.

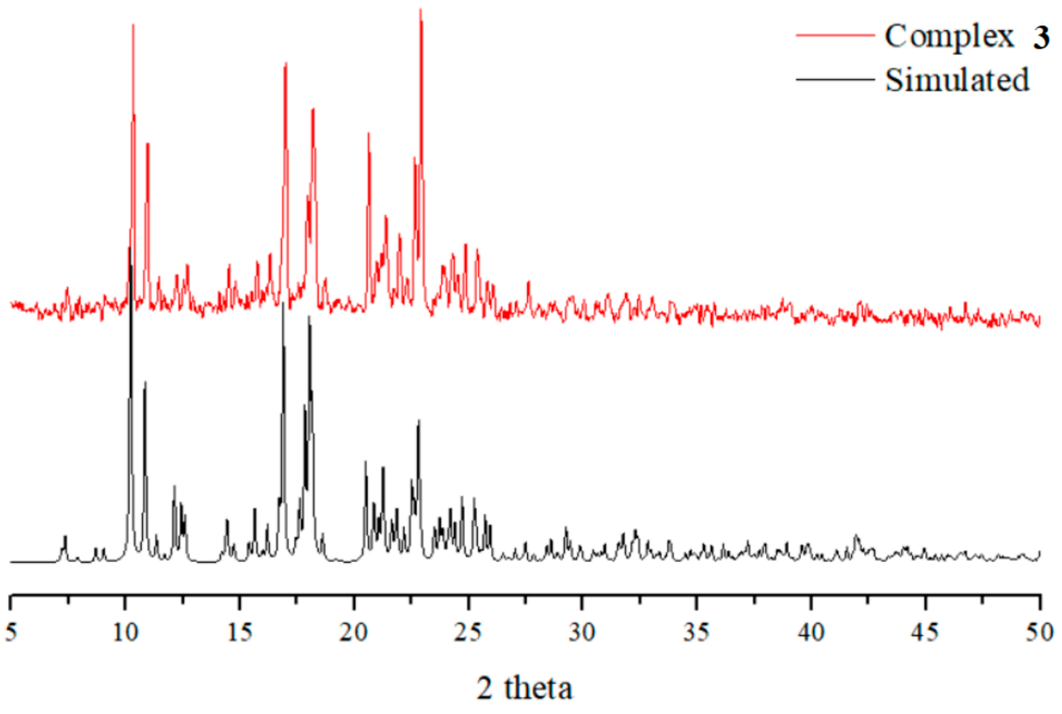

Figure S4. The PXRD patterns of complex **4**.

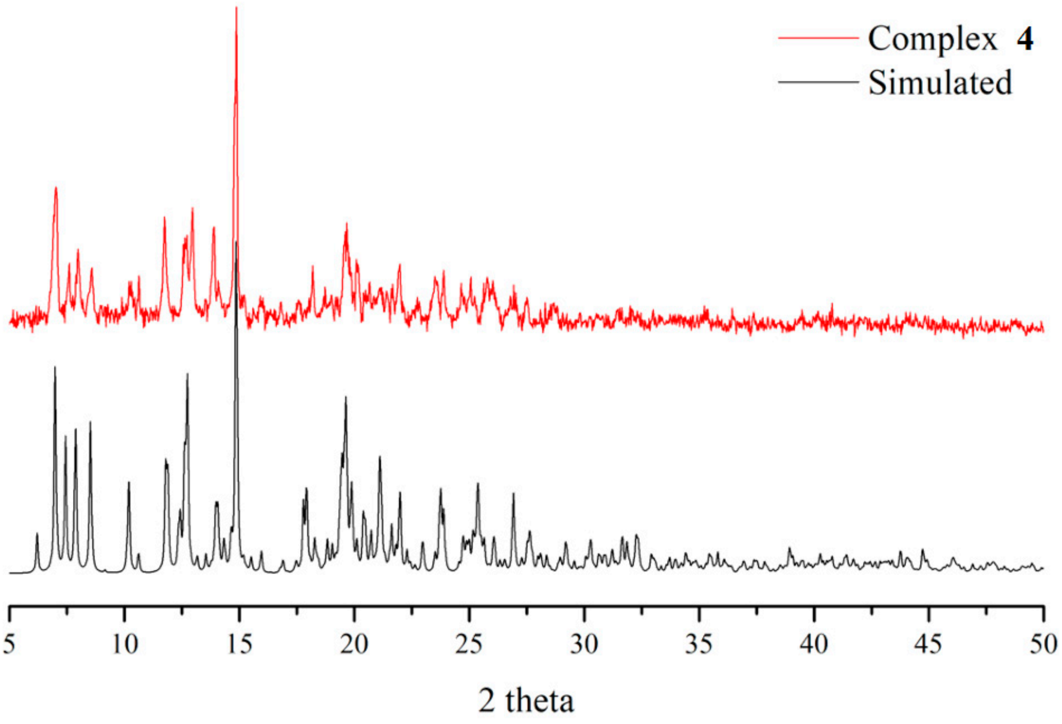

Figure S5. TGA curve of complex **1**.

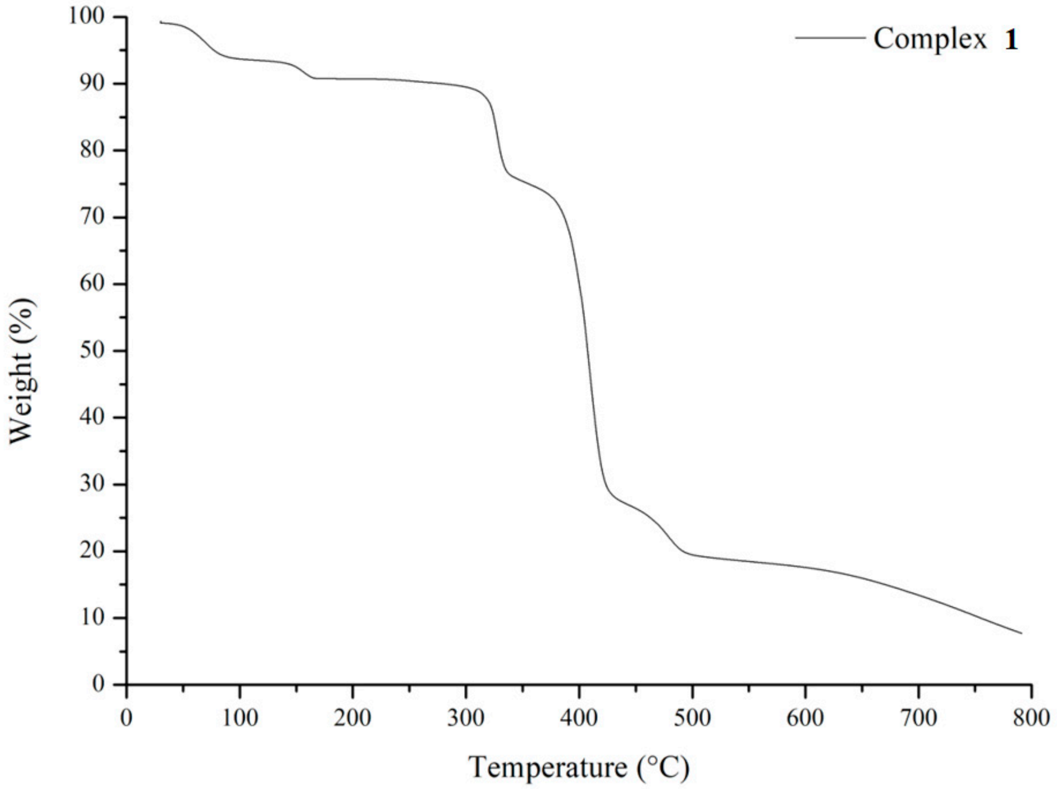

Figure S6. TGA curve of complex **2**.

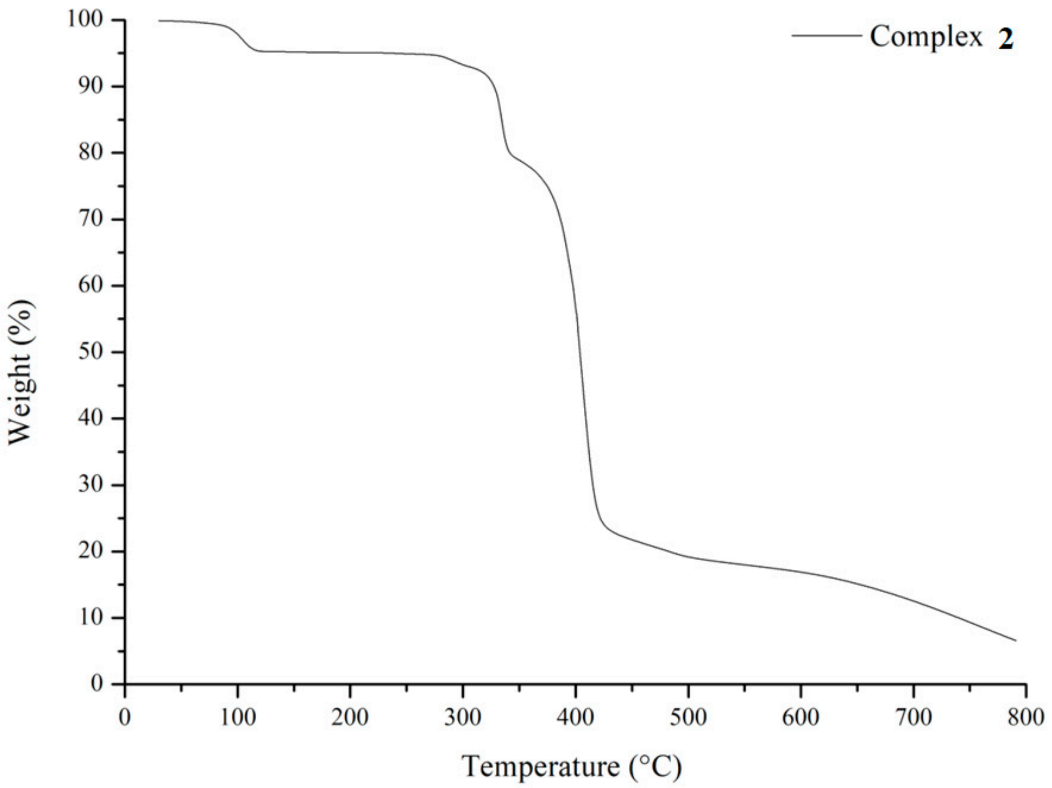

Figure S7. TGA curve of complex **3**.

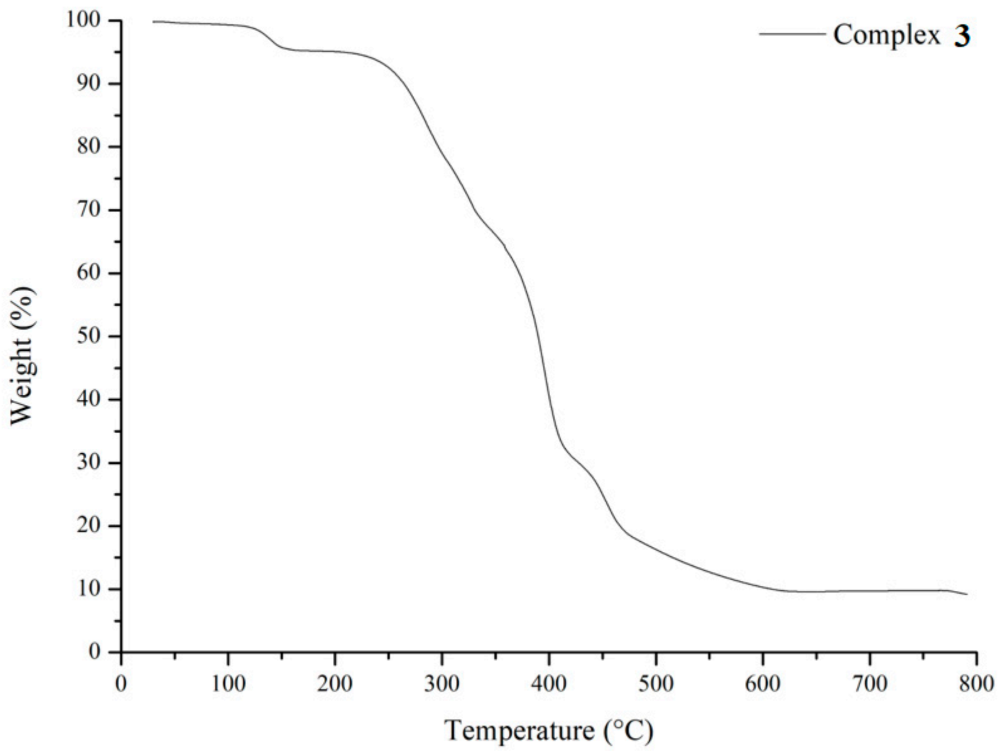

Figure S8. TGA curve of complex **4**.

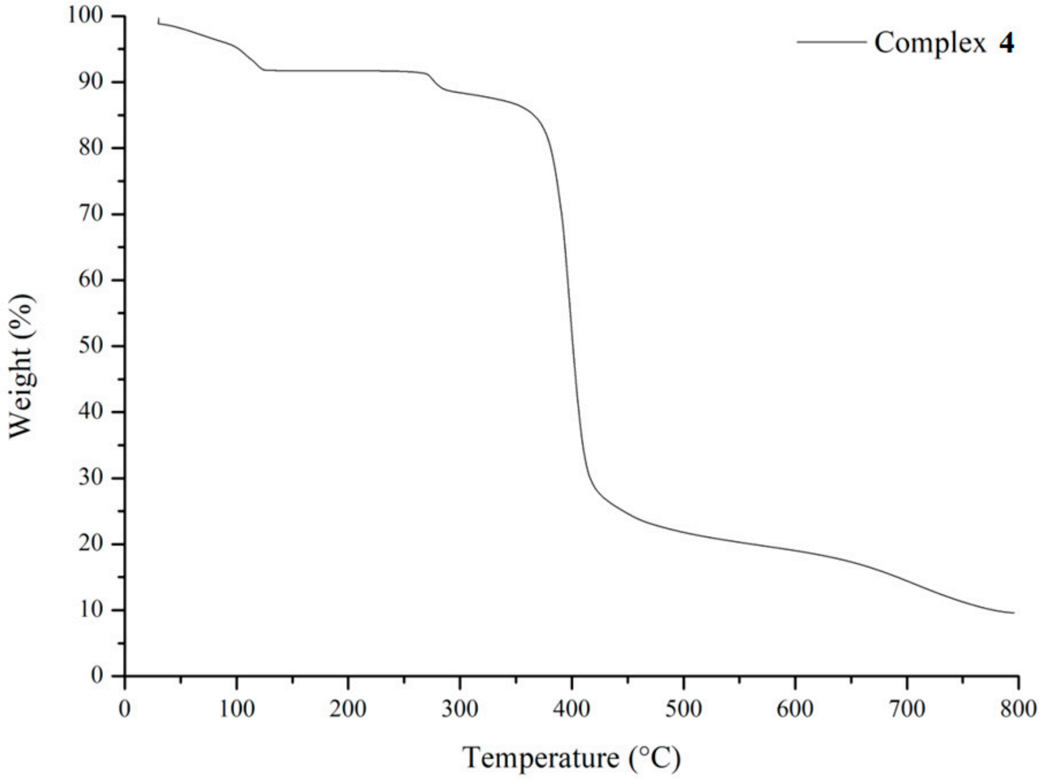

Figure S9. PXRD patterns of complex **1** immersed in a variety of organic solvents.

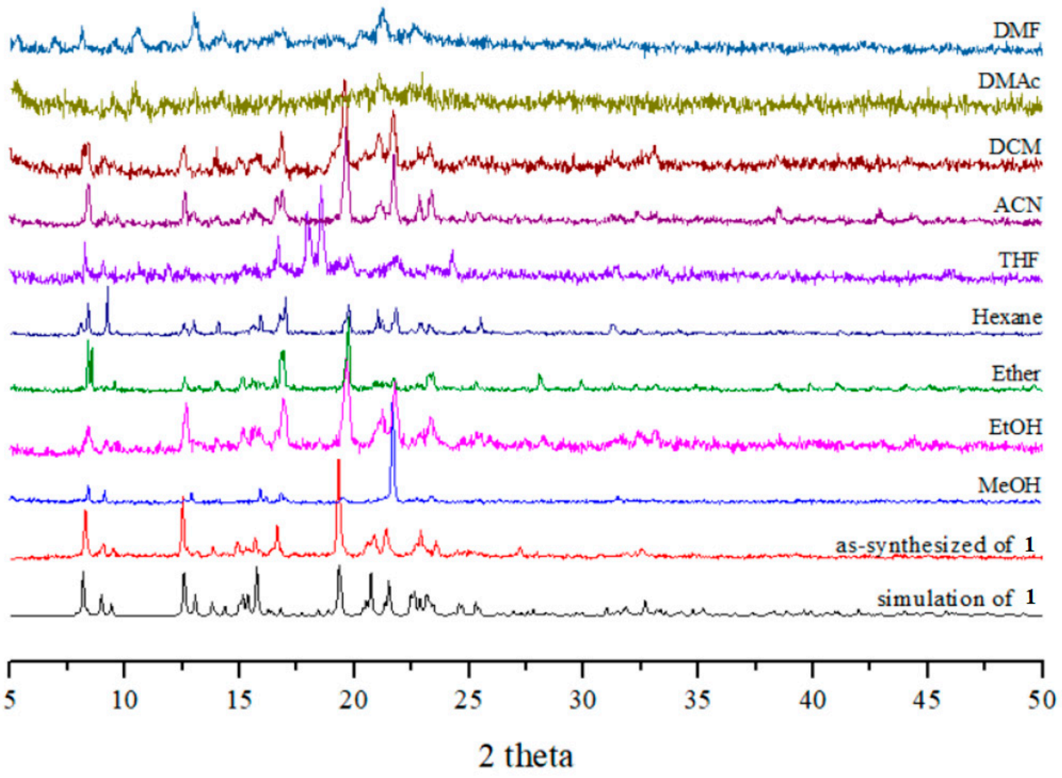

Figure S10. PXRD patterns of complex **2** immersed in a variety of organic solvents.

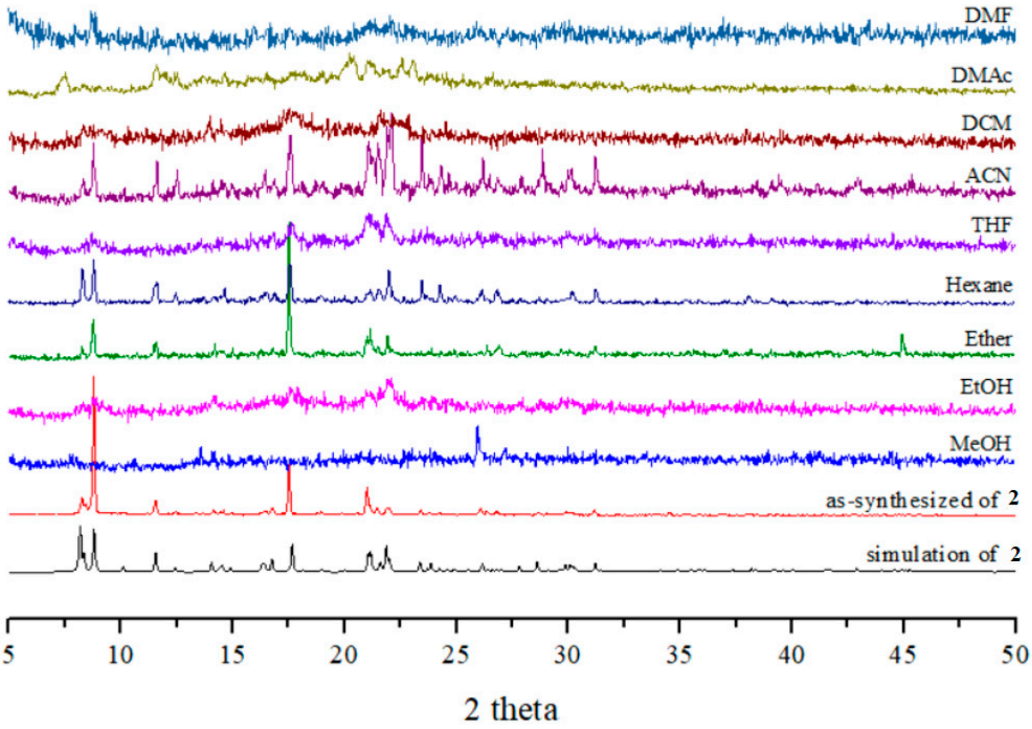

Figure S11. PXRD patterns of complex **3** immersed in a variety of organic solvents.

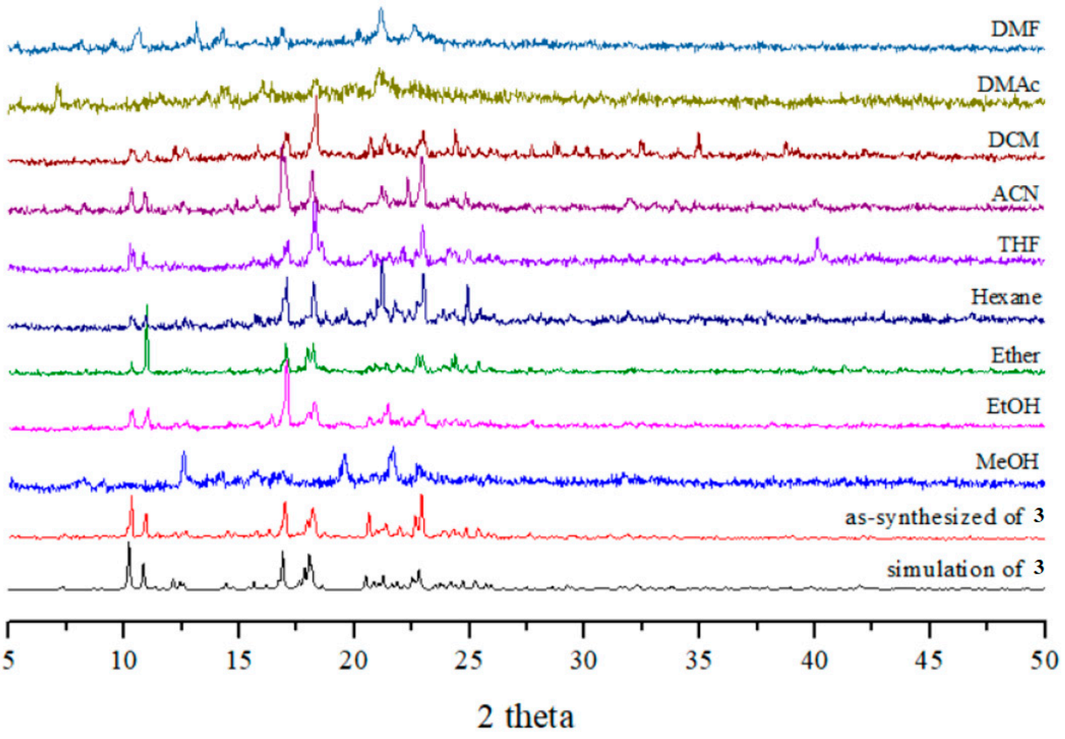

Figure S12. PXRD patterns of complex **4** immersed in a variety of organic solvents.

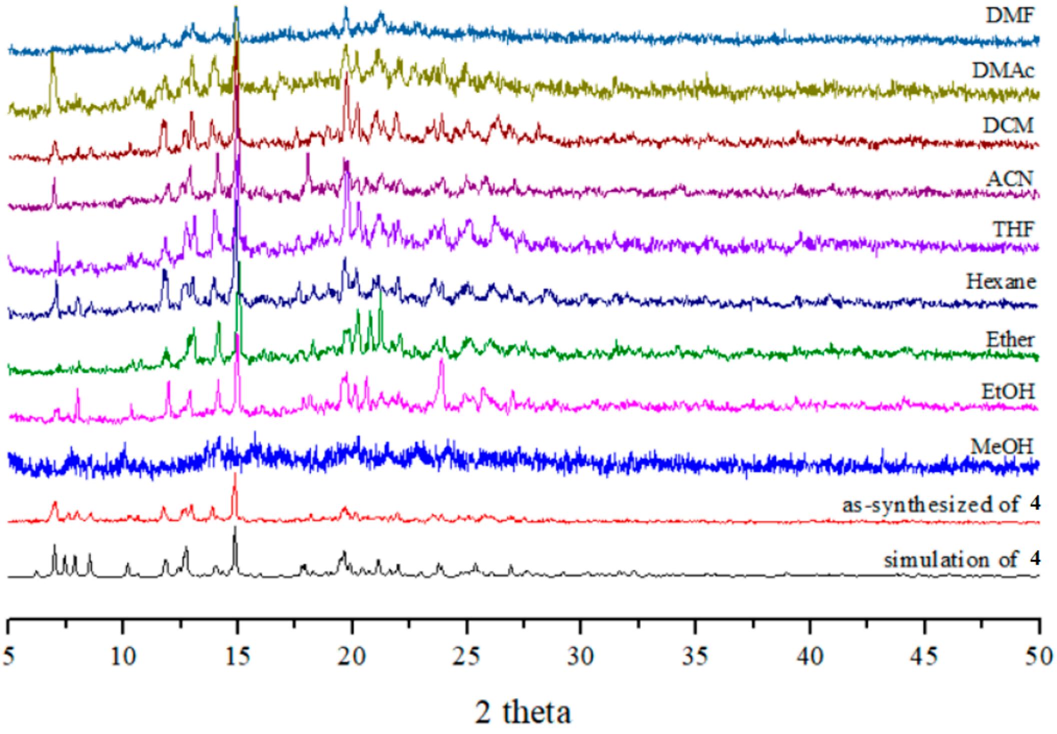

Figure S13. Photos of complexes **1** – **4** before and after heating.

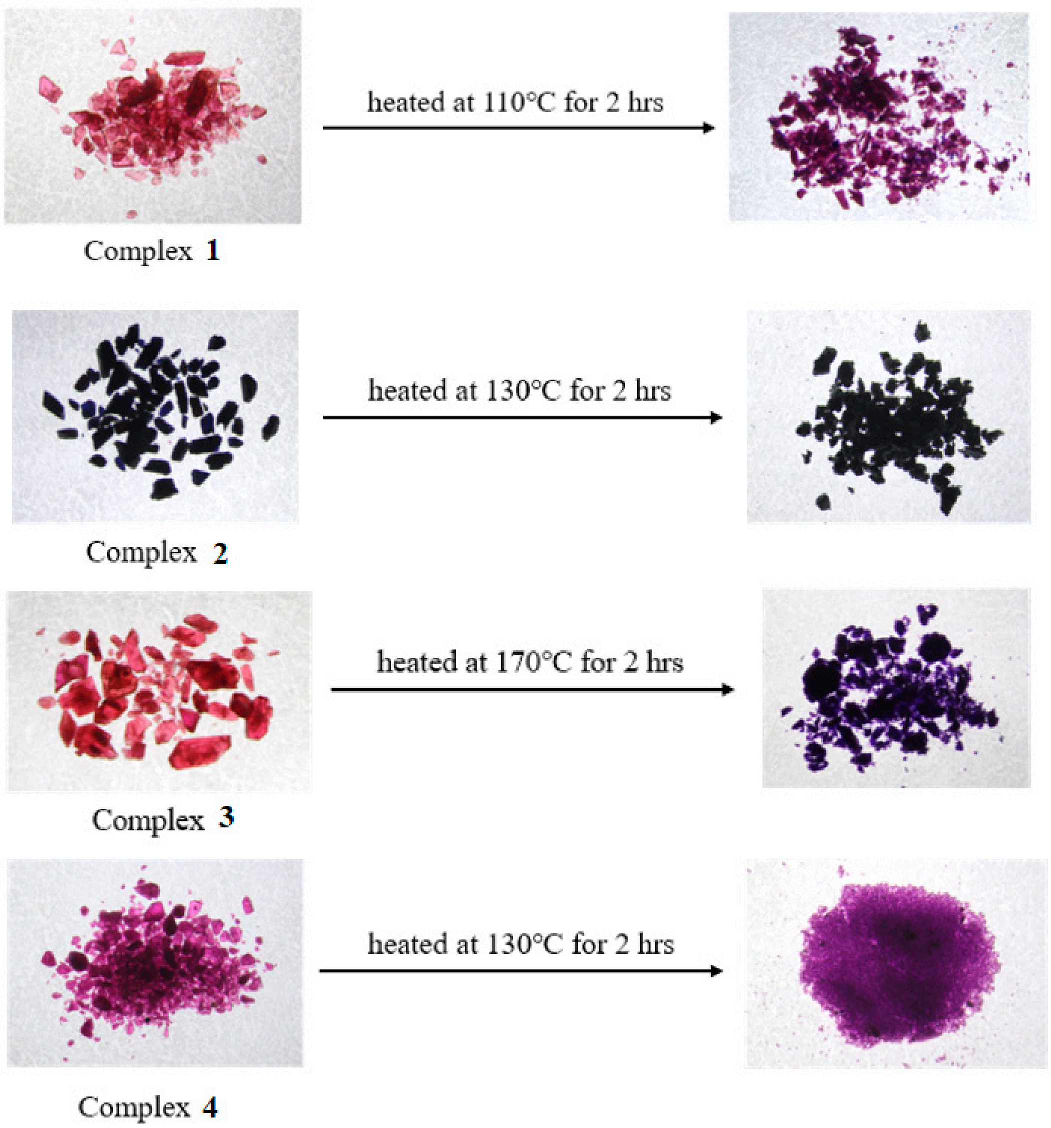

Figure S14. PXRD patterns of complex **1** heated at 110 °C for 2 hours and then immersed in a variety of organic solvents.

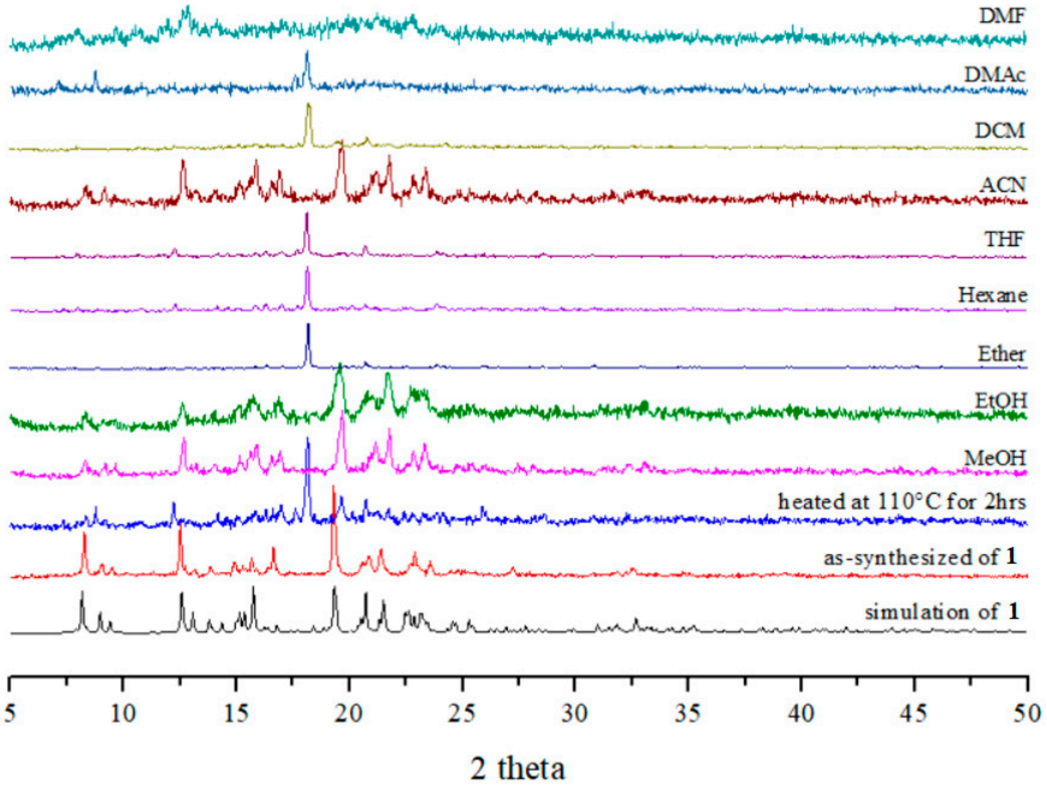

Figure S15. PXRD patterns of complex **2** heated at 130 °C for 2 hours and then immersed in a variety of organic solvents.

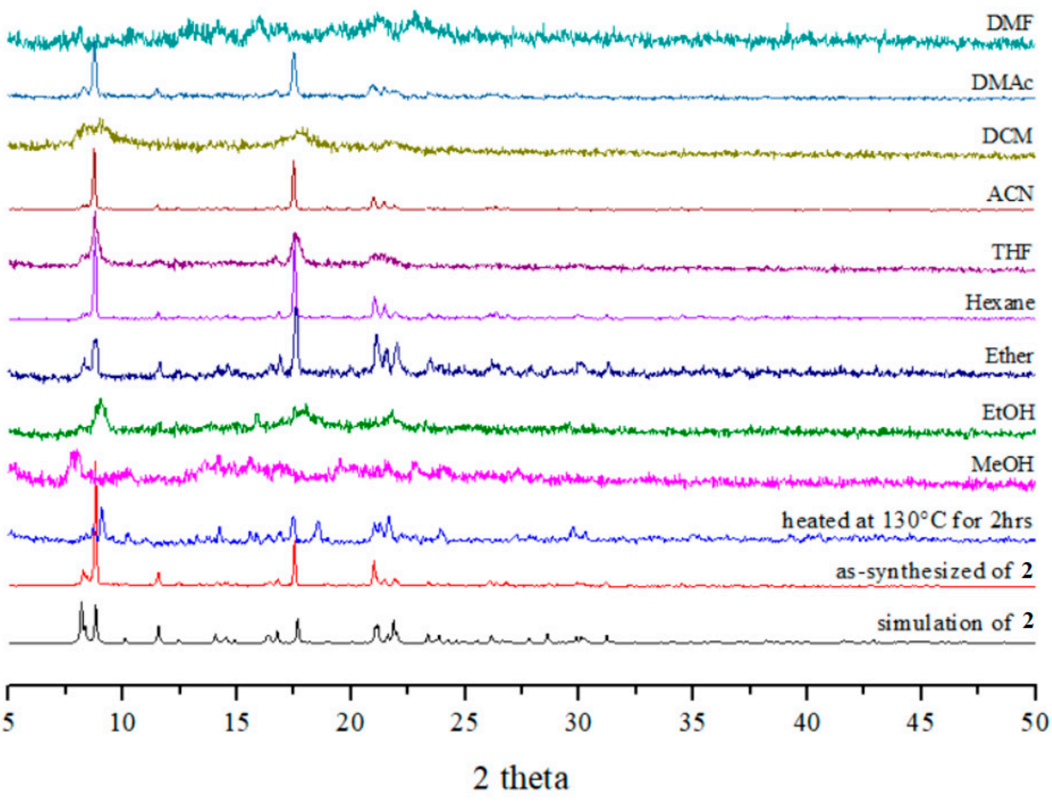

Figure S16. PXRD patterns of complex **3** heated at 170 °C for 2 hours and then immersed in a variety of organic solvents.

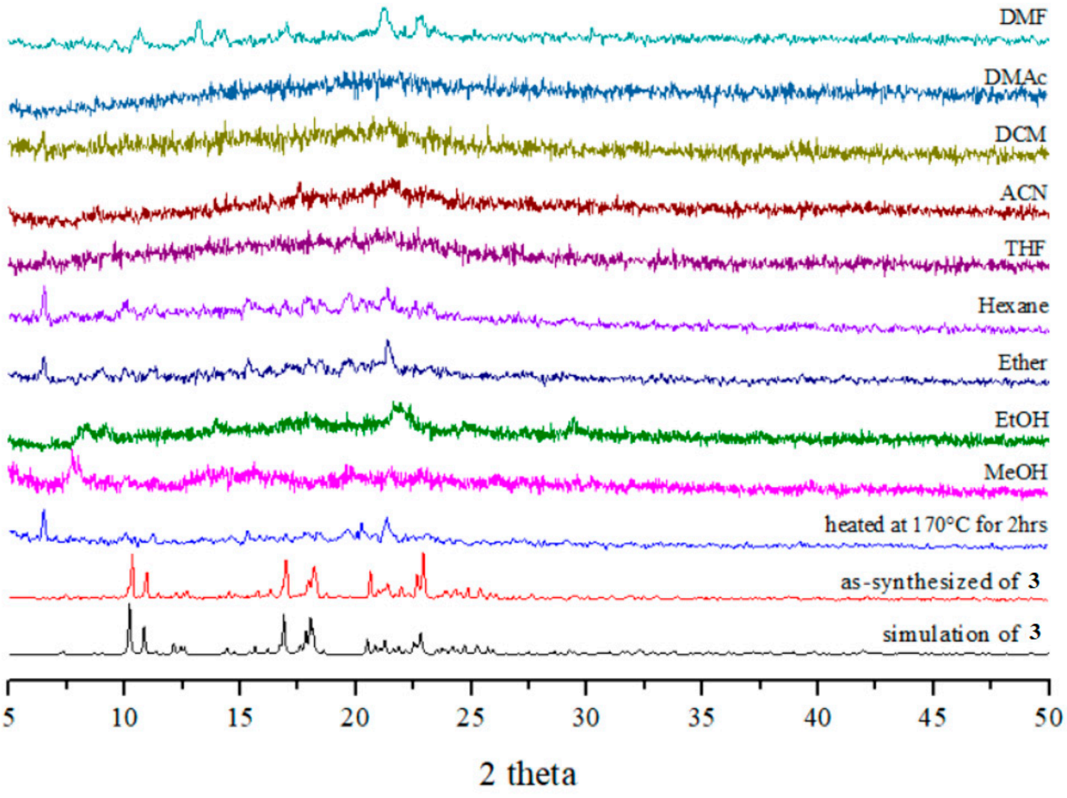

Figure S17. PXRD patterns of complex **4** heated at 130 °C for 2 hours and then immersed in a variety of organic solvents.

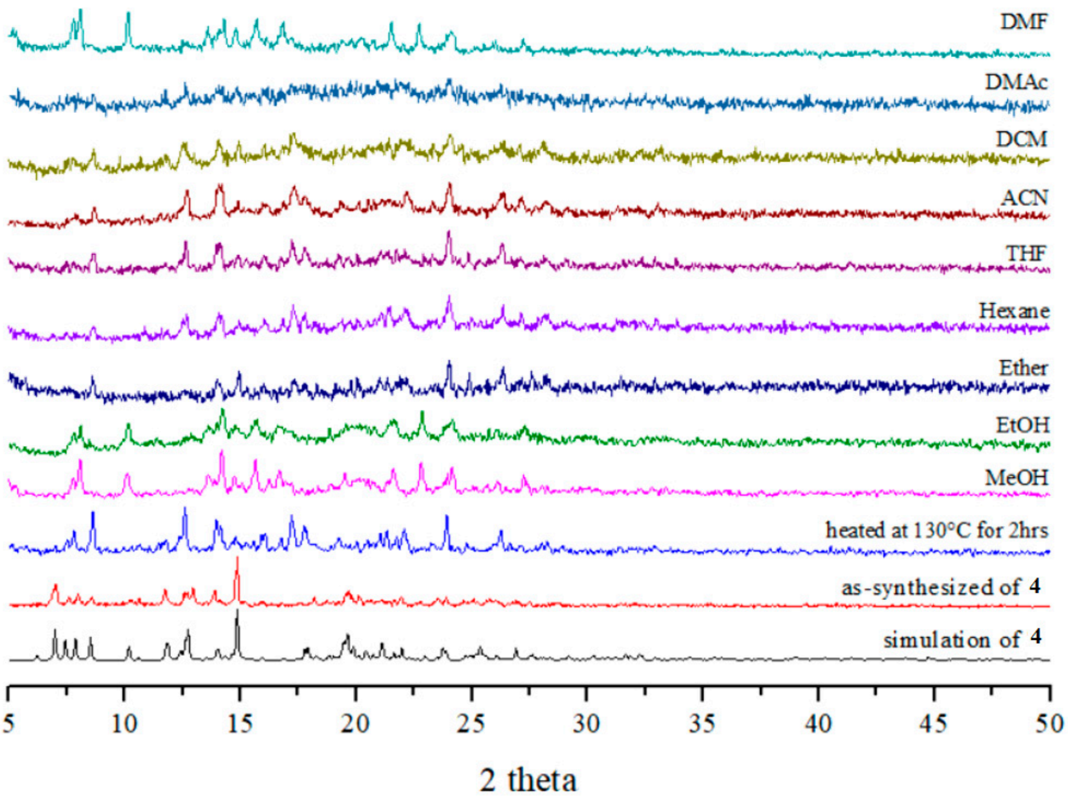

Figure S18. IR spectra for the structural transformations of **1** to **4** in water.

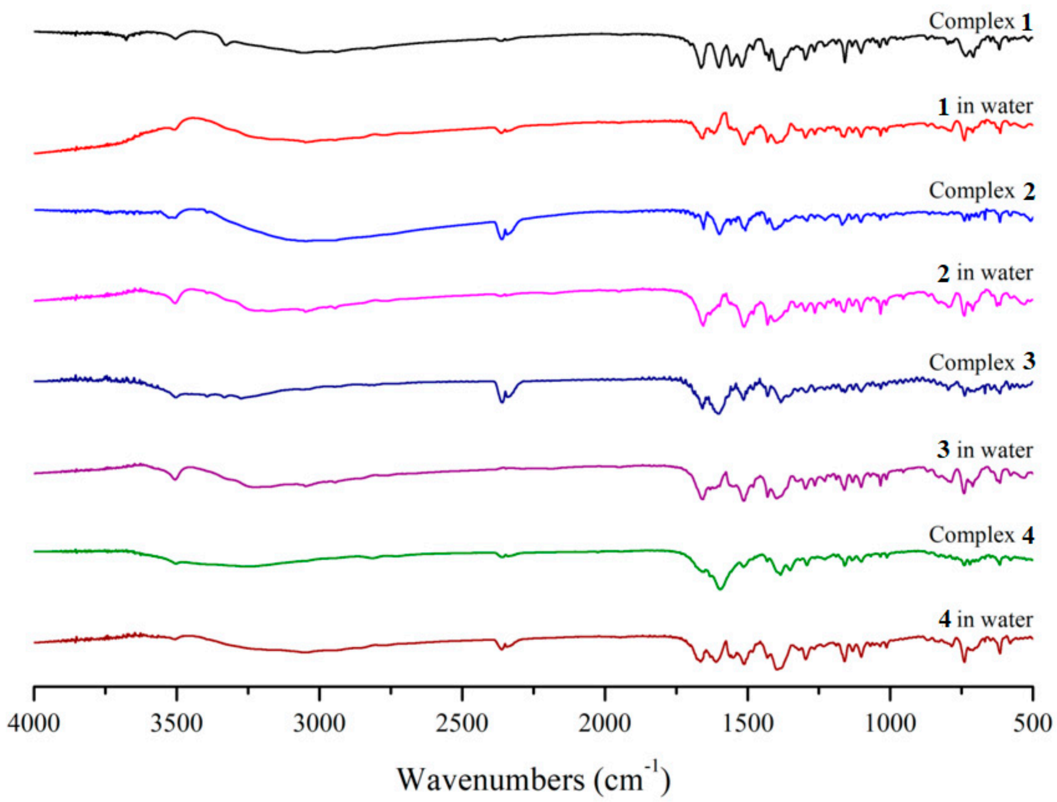

Figure S19. Infrared spectrum of complex **1.**

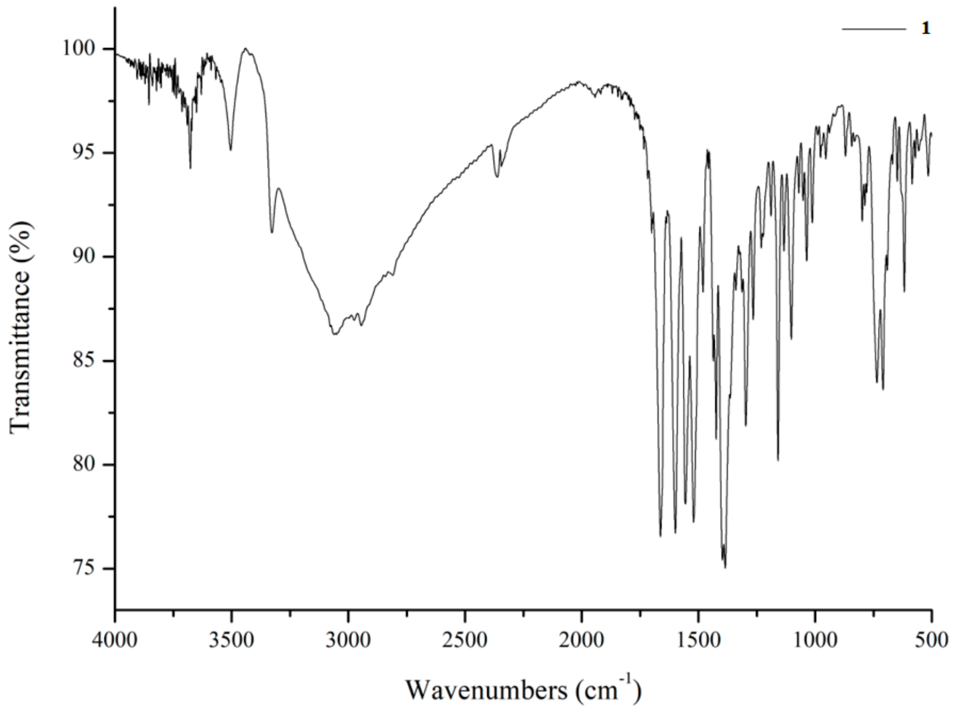

Figure S20. Infrared spectrum of complex **2.**

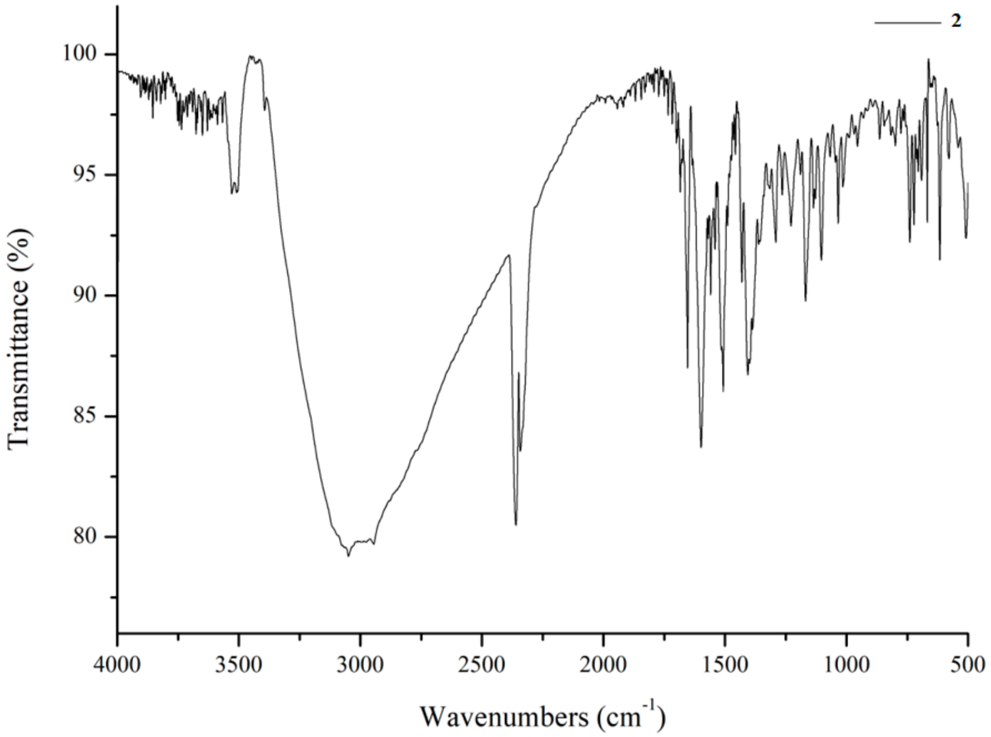

Figure S21. Infrared spectrum of complex **3.**

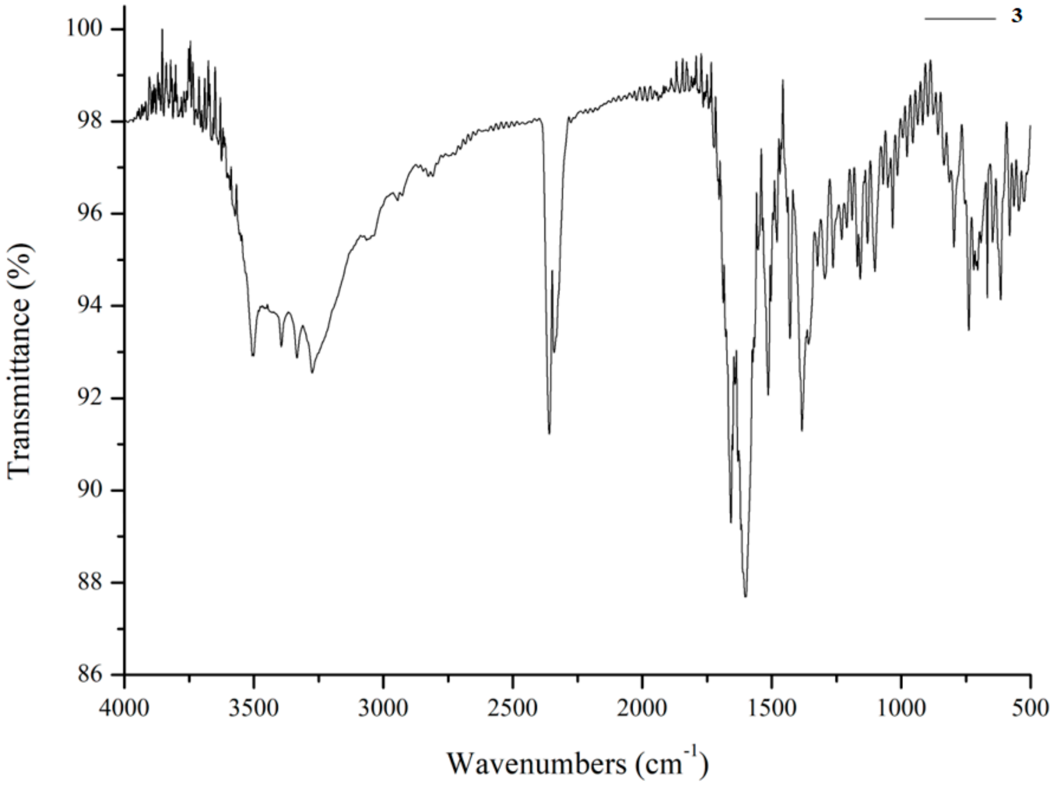

Figure S22. Infrared spectrum of complex **4.**

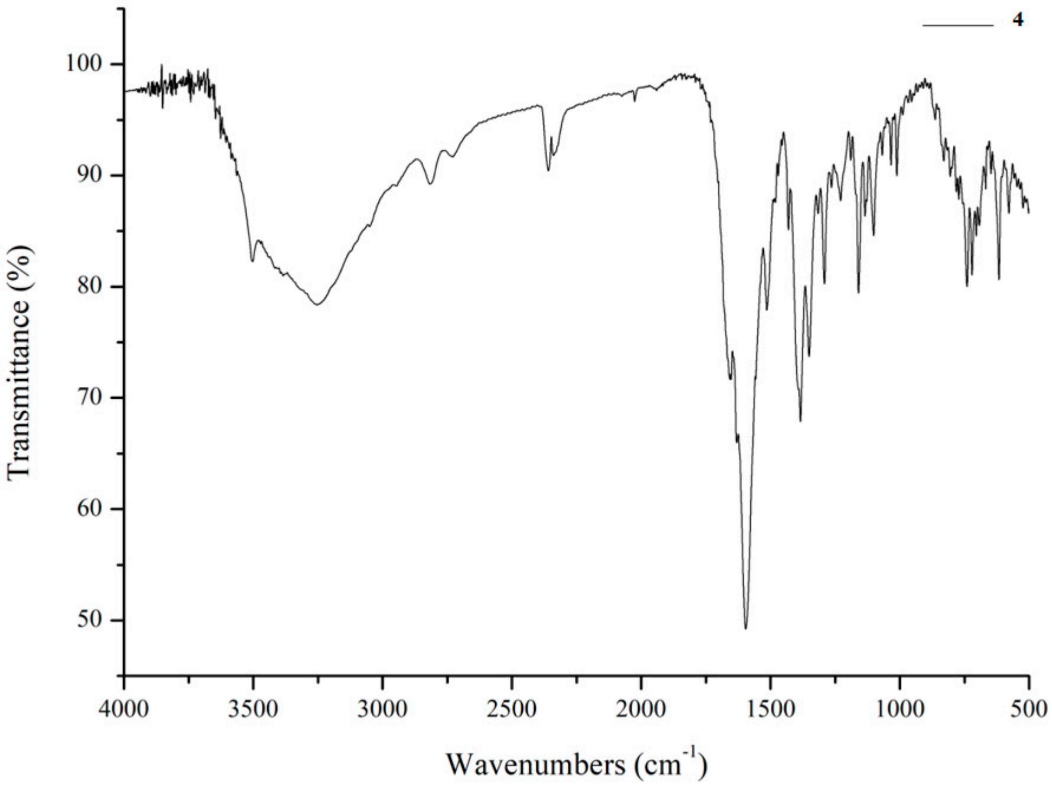

Supplement: Supplementary file 1 [file molecules-29-01748-s001.zip › Supplementary materials.pdf]
